# Genome-wide association mapping of growth dynamics detects time-specific and general quantitative trait loci

**DOI:** 10.1093/jxb/erv176

**Published:** 2015-04-28

**Authors:** Johanna A. Bac-Molenaar, Dick Vreugdenhil, Christine Granier, Joost J.B. Keurentjes

**Affiliations:** ^1^Laboratory of Plant Physiology, Wageningen University, Droevendaalsesteeg 1, 6708 PB Wageningen, The Netherlands; ^2^Laboratoire d’Ecophysiologie des Plantes sous Stress Environnementaux, UMR 759, Institut National de la Recherche Agronomique/Ecole Nationale Supérieure d’Agronomie, Place Viala, F-34060 Montpellier, Cedex 1, France; ^3^Laboratory of Genetics, Wageningen University, Droevendaalsesteeg 1, 6708 PB Wageningen, The Netherlands

**Keywords:** *Arabidopsis thaliana*, genome-wide association mapping, growth dynamics, GWAS, natural variation, PLA, plant phenotyping, projected leaf area, rosette growth.

## Abstract

Growth is a complex trait determined by the interplay between many genes, some of which play a role at a specific moment during development whereas others play a more general role. To identify the genetic basis of growth, natural variation in *Arabidopsis* rosette growth was followed in 324 accessions by a combination of top-view imaging, high-throughput image analysis, modelling of growth dynamics, and end-point fresh weight determination. Genome-wide association (GWA) mapping of the temporal growth data resulted in the detection of time-specific quantitative trait loci (QTLs), whereas mapping of model parameters resulted in another set of QTLs related to the whole growth curve. The positive correlation between projected leaf area (PLA) at different time points during the course of the experiment suggested the existence of general growth factors with a function in multiple developmental stages or with prolonged downstream effects. Many QTLs could not be identified when growth was evaluated only at a single time point. Eleven candidate genes were identified, which were annotated to be involved in the determination of cell number and size, seed germination, embryo development, developmental phase transition, or senescence. For eight of these, a mutant or overexpression phenotype related to growth has been reported, supporting the identification of true positives. In addition, the detection of QTLs without obvious candidate genes implies the annotation of novel functions for underlying genes.

## Highlight

Growth curve modelling and GWA mapping are combined to unravel the dynamic regulation of plant growth.

## Introduction

Plant growth is a dynamic process that is influenced by the many external and internal signals which the plant receives. For growth, a plant needs light and carbon dioxide to perform photosynthesis to produce sugars, which are the building blocks and energy source for many processes in the plant. In addition, the plant needs water and nutrients to be able to produce nucleotides, proteins, and metabolites. Transport of sugars and other essential molecules from source to sink is important during all stages of growth. Perturbation of these source–sink relationships by changes in the environment or due to the genetic composition of the plant may lead to changes in biomass accumulation. A better knowledge of the genetic factors that are involved in growth regulation would help in the understanding of the mechanisms underlying different growth patterns as observed in nature. Such dynamic patterns are better understood when growth and its regulation are studied over time, instead of at a single time point (Leister *et al.*, 1999; Granier *et al.*, 2006; Tessmer *et al.*, 2013).

Growth is orchestrated precisely and is controlled by many genes. The functional importance of most growth-related genes is not equal during all developmental stages and in all tissues, and many display specific temporal and spatial expression profiles (Schmid *et al.*, 2005). In addition, some genes play an essential role in the overall development of the plant, whereas others are mainly important if the plant has to cope with specific environmental conditions (Geng *et al.*, 2013). These tightly regulated genes form a robust network that enables the plant to complete its life cycle under many different circumstances.

Growth patterns of plants may differ widely between species (Westoby *et al.*, 2002), but also within the same species (Koornneef *et al.*, 2004; Alonso-Blanco *et al.*, 2009; Zhang *et al.*, 2012). Within species, the observed variation can be caused by differences in the local environment or can be due to natural genetic variation. This genetic variation is a result of random mutation and meiotic recombination, and can result in plants that, as a result of centuries of selection, are adapted to the local environment. The growth differences observed between and within species indicate that the regulation of growth is not only robust, but also genetically variable. Natural variation of growth within the same species can be used to search for genes regulating growth (Alonso-Blanco *et al.*, 2009). When growth phenotypes are determined in mapping populations, which are genotyped with many markers, linkage between genotypes and phenotypes can be identified by statistically testing the association between molecular markers and the observed phenotypes. Many mapping studies have been performed for plant growth and size resulting in the identification of many quantitative trait loci (QTLs) (Alonso-Blanco *et al.*, 1999; El-Lithy *et al.*, 2004; Lisec *et al.*, 2008; Chardon *et al.*, 2014). However, in those mapping studies, biomass was determined at the end of the experiment to evaluate the differences in growth (end-point measurements). As a result, only major players in the regulation of growth, such as genes that orchestrate the transition from a vegetative to a generative state (e.g. *FLC*) (Kowalski *et al.*, 1994) or genes related to dwarf growth (*erecta* locus or *ga20ox1*) have been cloned and confirmed (Komeda *et al.*, 1998; Barboza *et al.*, 2013). However, most of these major players were identified in experimental mapping populations in which only a few QTLs segregate or are artificially introduced (e.g. *erecta*). Additional players explaining a large part of the plant size variation observed in nature seem to be scarce. The mapping studies suggest that growth is a complex trait and that many genes are involved in the regulation of the accumulation of biomass. Genome-wide association (GWA) mapping studies might help to identify these genes because a much larger fraction of a species’ diversity is analysed.

Because growth is a dynamic process, the timing of the end-point measurement will greatly influence the outcome of the mapping (El-Lithy *et al.*, 2004). High-throughput automated phenotyping creates the possibility to follow the growth, or at least the two-dimensional (2D) expansion, of plants over time in a non-invasive way (Furbank and Tester, 2011). For plants with a 2D structure, such as rosettes of *Arabidopsis*, the challenge for high-throughput imaging is not only the capturing of pictures, but also the automatic image analysis. Different approaches dealing with this issue have been described: a pipeline to determine automatically rosette size (Hartmann *et al.*, 2011), rosette size and compactness (Arvidsson *et al.*, 2011), rosette shape (Camargo *et al.*, 2014), or rosette size accounting for leaf overlap (Tessmer *et al.*, 2013). The next challenge is how to use the additional information present in these time-series data for mapping purposes. Several ways in which data collected over time can be combined with mapping have been described, but no standard approach is agreed upon. The simplest approach is to treat data of different time points as unrelated traits and perform mapping for each time point separately, here referred to as univariate mapping of trait per time point (Moore *et al.*, 2013; Wurschum *et al.*, 2014). This approach resulted in the identification of time-specific QTLs for root bending upon a change in the direction of gravity in *Arabidopsis* (Moore *et al.*, 2013) and for plant height in wheat (Wurschum *et al.*, 2014). Another approach is to perform a two-step procedure. First a growth model is fitted to the growth data, after which the model parameters that describe the characteristics of growth are used in a standard mapping approach, here referred to as univariate mapping of model parameters (Heuven and Janss, 2010). This approach resulted in the detection of QTLs for the leaf elongation rate in maize (Reymond *et al.*, 2004). A similar approach was used to perform mapping on germination data, resulting in, for example, the detection of QTLs that are related to uniformity of germination (Joosen *et al.*, 2010). Finally, growth data collected over time can be analysed with multivariate mapping approaches (Ma *et al.*, 2002; Malosetti *et al.*, 2006; Yang *et al.*, 2011). The mapping power of multivariate approaches is higher, because they take into account that growth data collected over time and the derived parameters may be correlated, while univariate methods ignore this fact (Wu and Lin, 2006). Multivariate mapping can be done in a two-step approach in which a growth model is fitted to the growth data, after which the model parameters are used in a multivariate mapping approach (Korte *et al.*, 2012). This can also be applied in a one-step approach that uses one statistical model in which the molecular marker information and the parameters of a growth model are both included (Ma *et al.*, 2002). A multivariate approach was, for example, used to detect QTLs for progression of senescence over time in potato (Malosetti *et al.*, 2006), for leaf age based on the length and number of leaves emerging over time in rice (Yang *et al.*, 2011), and for stem diameter in poplar (Ma *et al.*, 2002). Univariate methods can be performed with standard mapping software packages, while multivariate mapping requires dedicated software (http://statgen.psu.edu/software/funmap.html; Korte *et al.*, 2012), which has hampered the adaptation of multivariate mapping by a broad community. Although each of the described methods has its own drawbacks, they clearly show that mapping of data collected over time results in the identification of QTLs that would not have been detected if only single-point measurements were used as input for the QTL analyses.

Here, a series of analyses that enable the observation of growth dynamics by automatic imaging are described. It will be shown that top-view imaging of *Arabidopsis* plants in combination with high-throughput image analysis can be used to follow rosette growth over time in a large and diverse population of natural accessions. It will further be shown that comparison of accessions demonstrating a large variation in developmental rate and in plant size can be done by modelling of growth. In addition, GWA mapping on temporal plant size data was performed using univariate and multivariate mapping approaches. Time-specific growth QTLs were detected by performing univariate GWA mapping for each time point separately, whereas general QTLs related to growth rate during the course of the whole experiment were identified by performing univariate and multivariate GWA mapping on the growth model parameters. Finally, candidate genes involved in the regulation of growth could be indicated.

## Materials and methods

### Plant material

A collection consisting of 324 natural accessions of *Arabidopsis thaliana* was used to investigate the growth of rosettes over time (Supplementary Table S1 available at *JXB* online). These accessions were selected to capture most of the genetic variation present within the species (Baxter *et al.*, 2010; Li *et al.*, 2010; Platt *et al.*, 2010). Each accession was genotyped with ~215 000 single nucleotide polymorphism (SNP) markers (Col versus non-Col) (Kim *et al.*, 2007).

### Experimental set-up

The PHENOPSIS phenotyping platform was used to perform the experiments (Granier *et al.*, 2006). The climate conditions within the growth chambers of PHENOPSIS are precisely regulated, preventing differences in growth because of position in the chamber. The plants were grown in four adjacent independent experiments each containing 84 accessions (four rounds) (Supplementary Table S1 at *JXB* online). Each experiment contained three replicates of a completely randomized block (three blocks), including four reference accessions grown in each experiment: Col-0 (CS76113), KBS-Mac-8 (CS76151), Lillo-1 (CS76167), and Wc-2 (CS28814). Note that the reference accessions (checks) were used to correct for round effects, as all other accessions were only analysed in one of the four experiments. Cylindrical pots (9cm high, 4.5cm in diameter) filled with a mixture (1:1, v/v) of a loamy soil and organic compost were used, and the seeds (at least two per pot) were sown directly on the soil. The seeds and pots were subjected to cold treatment (4 °C) directly after sowing. To enable harvesting of the rosettes within a time frame of 1.5h, the three blocks were transferred from the cold to the growth chamber (PHENOPSIS, 16h light, 125 μmol s^–1^ m^–2^, 70% humidity, 20/18 °C) on sequential days, 4, 5, or 6 d after sowing. The day the plants were transferred to PHENOPSIS was denoted as day 1. The water content of the soil was kept at 0.35–0.40g H_2_O g^–1^ dry soil by robotic weighing and watering the pots twice a day. After 2 weeks, the plants were thinned to one plant per pot.

A separate experiment was performed to determine whether the accessions were summer or winter annuals. All accessions (three replicates) were grown on rockwool blocks in the greenhouse and were watered regularly. The flowering time of the first replicate of each accessions was recorded. Accessions that flowered within 75 d were called summer annuals; accessions that did not flower within this period were called winter annuals.

### Determination of rosette growth traits

All plants were inspected daily for visible signs of bolting, and bolting dates were recorded (Supplementary Table S1 at *JXB* online). At day 24, the largest leaf of each plant was harvested. The fresh weight (FW) and dry weight (DW) of the leaves were determined to calculate the water contents (WCs) by WC=(FW–DW)/FW. At day 28, the rosettes were harvested and the FWs were determined. Rosette growth was monitored by taking pictures from above twice a day. These pictures were processed in ImageJ using the macros developed for PHENOPSIS. All pictures and the ImageJ macros are publically available on PHENOPSISDB (Fabre *et al.*, 2011; http://bioweb.supagro.inra.fr/phenopsis). The projected leaf area (PLA) of each plant was determined semi-automatically on days 8, 11, 14, 16, 18, 20, 22, 24, 25, 26, 27, and 28. When more than one plant was present in a pot before thinning, the largest one close to the middle of the pot was taken for analysis. For each individual plant, the growth, based on PLA, was modelled using three functions: two functions describing indeterminate growth by an exponential curve, Expo1 and Expo2; and one function describing determinate growth by a S-curve, Gom (Table 2). The optimal parameter values were estimated using the Growth Fitting Toolbox^TM^ of MATLAB with the following settings. Expo1: optimization algorithm, ‘Trust-Region’; fitting method, non-linear least square; bounds, *r* [0, Inf]. Expo2: optimization algorithm, ‘Trust-region’; fitting method, non-linear least square; bounds, *A*_0_[0,Inf], *r*[0,Inf]. Gompertz: optimization algorithm: ‘Levenberg–Marquardt’; fitting method, robust linear least square, using bisquare weights; bounds, *A*_max_[0,20000], *b*[0,60], *r*[0,Inf]. Goodness of fit indicators (SSE, *r* ^2^, and RMSE) and 95% confidence intervals of the parameters of all three models were calculated in MATLAB (Supplementary Table S2). Based on these data and the principle of parsimony, Expo2 was chosen as the best model and was therefore used for further analysis; fits with *r* ^2^<0.9 (11 out of 965) were excluded from further analysis. The removal of the largest leaf from day 24 onwards was not corrected for when the fresh weight of the rosette and the model parameters were determined because plants rapidly compensated for this loss.

### Statistical analysis

GWA mapping was performed on the FW at day 28, PLA at days 8, 11, 14, 16, 18, 20, 22, 24, 25, 26, 27, and 28, and the estimated model parameters of Expo2. For all traits, adjusted means for each accession were obtained with GenStat by fitting the following mixed model:

$$\begin{array}{l} \text{Trait }\left(\begin{array}{l} \text{FW},\text{ PLA},\text{ or Expo2 }\\ \text{model parameters} \end{array}\right)=\mu +\text{Check}+\text{Accession}\left(\text{Round}\right)+\text{Round}\\ \;+\text{Round}\times \text{Check}+\text{Block}\left(\text{Round}\right)\\ \;+\text{Block}\left(\text{Round}\right)\times \text{Check}+\text{error} \end{array}$$

where Check refers to a factor in which reference accessions Col-0 (CS76113), KBS-Mac-8 (CS76151), Lillo-1 (CS76167), and Wc-2 (CS28814) were distinguished from the other accessions, Accession refers to the 324 different accessions analysed, Round is a factor with four variables corresponding to the four experiments of 84 accessions, and Block is a factor with three variables corresponding to the three replicates within each round. The terms Checks and Accessions within Round were assumed fixed to obtain Best Linear Unbiased Estimates (BLUEs), and all the remaining terms were considered random effects as they are all essentially different sources of experimental error due to Round, Blocks within Rounds (and the interaction with check genotypes), and residual variation. GWA mapping was performed on the predicted means using the EmmaX software package for R, which is based on Kang *et al.* (2010). A mixed model was used that corrects for population structure, based on the kinship matrix of all SNPs. SNPs with a minor allele frequency <0.05 were excluded from the analysis. The parameters ‘*A*_0_’ and ‘*r*’ of model Expo2 were also mapped together using a Multi Trait Mixed Model (MTMM) approach (Korte *et al.*, 2012; El-Soda *et al.*, 2015). Pearson correlations were used to determine correlations between data series. To calculate the correlation between traits, the corrected means were used. Broad-sense heritabilities were obtained with GenStat by fitting the same model as above. This time the term Check was assumed fixed and all the remaining terms were considered random effects. Broad-sense heritability at the mean level was calculated as: *H* ^2^=*V*_g_/(*V*_g_+*V*_e_/*r*), where *V*_g_ is the genetic variance (Accessions), *V*_e_ is the error variance, and *r* is the number of replicates for each accession in each experiment (*r*=3).

Differences in FW between the rosettes of plants that were bolting at day 28 and plants that were still vegetative, and between summer and winter annuals were determined using a *t*-test assuming non equal variances and α=0.05.

### Additional analyses

For each of the candidate genes, the annotations and gene ontology (GO) terms were retrieved from TAIR10 (arabidopsis.org).

## Results and Discussion

### Capturing the dynamics of growth by top-view imaging

**Fig. 1.**
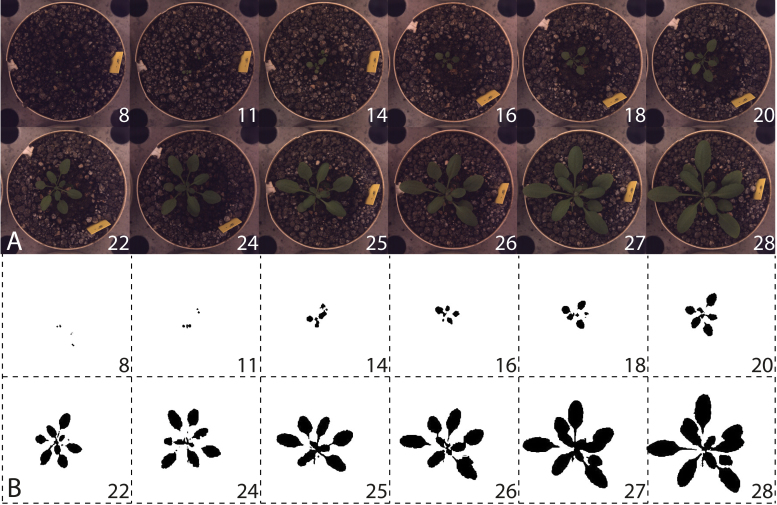
(A) Images of one of the replicates of CS28014 (Amel-1), a representative accession, at all time points included in the analyses. (B) Pictures processed by ImageJ to determine the projected leaf area (PLA). Pictures were segmented based on colour, saturation, and brightness, and thereafter made binary. Particles which were too small (<120 pixels) were excluded from the analysis. In the images of days 8, 11, and 14, more than one plant is present, but only the remaining one (days 16 and onwards) is taken into account for PLA determinations.

A large-scale experiment was performed in the plant phenotyping platform PHENOPSIS (Granier *et al.*, 2006). A total of 324 natural accessions of *A. thaliana* were grown and their rosette sizes were monitored over time by capturing top-view pictures daily (Supplementary Table S1 at *JXB* online). The plant architecture of the vegetative stage of *Arabidopsis* makes this species very suitable for top-view imaging. Because the rosette grows in a horizontal plane, it can be approached as a 2D structure the size of which can be determined accurately from top-view images. Top-view imaging of *Arabidopsis* rosettes was first reported in the 1990s (Leister *et al.*, 1999), but became suitable for large populations only recently due to advances in the automation of image analysis (Berger *et al.*, 2010; Arvidsson *et al.*, 2011; Tessmer *et al.*, 2013). Although at the moment low-cost, high-throughput methods are available to determine the genome of an organism and genetic information is available for many species and for many mutants and natural accessions, the plant science community lags behind in the high-throughput measurements of phenotypes (Houle *et al.*, 2010). In this experiment, top-view imaging in combination with high-throughput image analysis allowed the determination of the rosette size of plants of 324 accessions in triplicate at 11 time points during growth. PLA was determined from day 8 onwards and the experiment was ended before too many leaves were overlapping (Fig. 1). On day 8, all seeds had germinated, the cotyledons were unfolded, but the first true leaves were not yet visible. As the growth rate increased during the course of the experiment, the interval between the time points of PLA determination was decreased, from a 3 d interval in the second week to a 1 d interval in the fourth week, to ensure that dynamics in growth were accurately captured. Because diurnal leaf movement was observed, PLA was always determined within 2h after the start of the light period. This analysis is one of the first steps in the detailed characterization of the phenomes of these natural accessions (Furbank and Tester, 2011). Similar approaches can also be used in the future to characterize further the phenomes of these natural accessions by performing similar experiments when plants are grown in different and possibly less favourable conditions, such as short days or under abiotic or biotic stress. For much smaller sets of accessions, similar experiments have previously been performed, but to be able to use the phenotypes in mapping studies much larger populations need to be screened (El-Lithy *et al.*, 2004; Granier *et al.*, 2006). Growth was determined not only by differences in PLA over time, but also at the end of the experiment by measuring the FW of the rosettes.

**Fig. 2.**
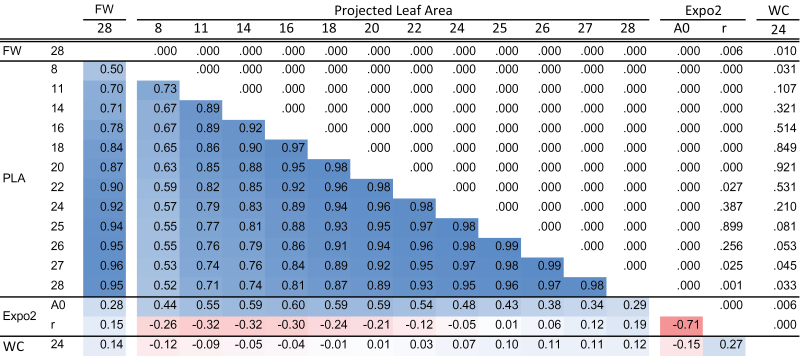
Pearson correlations between fresh weight of rosettes (FW) at the end of the experiment (day 28), projected leaf area (PLA) over time (day 8 till 28), and parameters ‘*r*’ and ‘*A*_0_’ of the growth model Expo2. *r* ^2^-values are given in the left lower part of the figure, whereas corresponding *P*-values are given in the right upper part of the figure. Blue and red indicate positive and negative correlations, respectively. The stronger the intensity of the colour, the stronger the correlation.

For PLA and FW, large natural variation was observed, 28–70% of which could be explained by genetic differences (Table 1). Broad-sense heritability (*H* ^2^) of PLA increased over time (Table 1), most probably because determination of the PLA of small plants was less accurate than that of larger plants. These data demonstrate that top-view imaging of *Arabidopsis* is a powerful method to compare plant size and growth rate in large panels of plants which differ not only in size but also in developmental traits such as flowering time (Li *et al.*, 2010), number of leaves, and leaf emergence rate (Granier *et al.*, 2006; Tisné *et al.*, 2010). FW at the end of the experiment correlated positively with PLA at the end of the experiment (*r* ^2^=0.95), as shown earlier (Leister *et al.*, 1999). This high correlation is also reflected in almost equal *H* ^2^ of FW and PLA at day 28 (*H* ^2^=0.69 and *H* ^2^=0.70, respectively). FW also correlated with PLA in weeks 2 and 3 (Fig. 2). In the last week of the experiment leaves started to overlap, and variation for this trait was observed between accessions. Despite this increase in overlap over time, the correlation between FW on day 28 and PLA on the sequential measuring dates increased over time, reaching the highest correlation on day 27 (*r* ^2^=0.96). This correlation suggests the existence of general growth factors whose effects are visible at the phenotype level during a large part of the plant’s life cycle. Seedling size at day 8, when the cotyledons are unfolded but the first true leaves are not yet visible, is for a large part determined by seed size, germination rate, and the capacity of the seedling to establish. The correlation of PLA during the experiment also suggests that the effects of genes involved in the regulation of these processes are visible at the phenotype level when seedlings develop into plants with many leaves. The water status of the plant was evaluated by the determination of the WC of the largest leaf on the 24th day. A proper water status is important for the plant to maintain growth. WC was high for all plants (between 0.85 and 0.95), indicating that in the conditions used here the water status was not limiting for growth. This corresponds to small variation in WC observed in a collection of 20 accessions (El-Lithy *et al.*, 2004). Significant but very weak correlations were observed between WC and *A*_0_, *r*, FW, and PLA on days 8, 27, and 28, whereas the correlation between WC and plant size on other days was not significant. Because of the low variation observed, WC did not play a prominent role in determining growth differences in this experiment. Because PLA of the rosette was on average doubled during the last 4 d of the experiment, it was decided not to correct for the absence of the largest leaf. In the growth curve of some accessions between day 24 and 25, a dip is observed; however, for many accessions, this dip was hardly visible, suggesting a huge compensation investment in the growth of the remaining leaves. Without correction for the absence of the leaf, the growth modelling resulted in very reliable curve fits for Expo2 and Gom, indicating that the growth rate was hardly influenced by the removal of the largest leaf.

**Table 1.** Natural variation and broad-sense heritabilities for growth traits and growth model parameters

		Days	Average	Minimum	Maximum	SD	*H* ^2^
FW (g)		28	0.26	0.01	0.74	0.12	0.69
PLA (mm^2^)		8	6	1	42	3	0.28
		11	15	3	48	7	0.52
		14	36	4	153	16	0.51
		16	61	6	185	29	0.52
		18	110	8	362	54	0.55
		20	188	21	590	91	0.55
		22	314	14	986	153	0.59
		24	495	32	1438	235	0.62
		25	520	27	1627	257	0.61
		26	640	40	2041	312	0.62
		27	769	43	2450	362	0.65
		28	911	52	2832	417	0.70
Expo2:	*A*_0_		5.33	0.21	47.22	4.49	0.63
	*r*		0.19	0.10	0.31	0.02	0.28

FW, fresh weight of rosettes (g); PLA, projected leaf area of rosette (mm^2^); Expo2, growth model using an exponential function with two parameters, *r* (growth rate) and *A*_0_ (initial size and magnification factor); days, days after transfer from cold to the climate room; Average, Minimum, Maximum, SD, average value, minimum value, maximum value, and standard deviation observed for the indicated trait on the indicated date; individual plants are used instead of averages per accession; *H* ^2^, broad-sense heritability, *H* ^2^=*V*_g_/(*V*_g_+*V*_e_/*r*), where *V*_g_ is the genetic variance, *V*_e_ is the error variance, and *r* is the number of replicates for each accession in each experiment (*r*=3).

Natural variation in bolting time was also observed among the accessions analysed (Supplementary Table S1 at *JXB* online). Plants that started bolting before the end of the experiment were significantly larger than vegetative plants. A similar pattern was observed when plants were classified as winter or summer annuals, the first of which require vernalization to flower. Summer annuals, many of which flowered at day 28, were significantly larger than winter annuals, none of which was flowering in this experiment. Winter annuals germinate in autumn and survive winter as small plants, for which fast growth is not a priority (Gazzani *et al.*, 2003; Grennan, 2006). Summer annuals, on the other hand, germinate in spring and have to finish their life cycle before the dry and hot summer period, and fast growth might, therefore, be an advantage. When grown in optimal growing conditions, these properties may result in the observed differences in size.

### Comparison of models to describe early vegetative growth

**Fig. 3.**
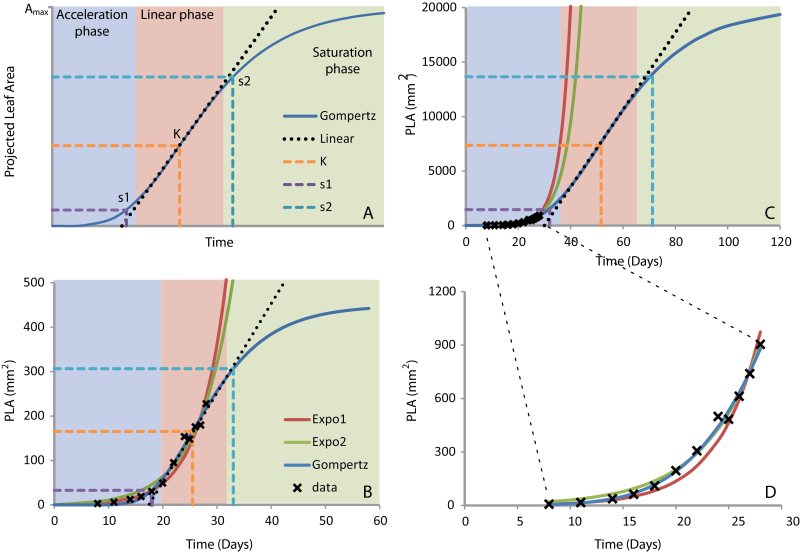
Modelling of growth using three mathematical functions, Expo1, Expo2, and Gom (see Table 2 for details). (A) S-curve as a model for determinate growth consisting of three phases: the acceleration phase, the linear phase, and the saturation phase. The S-curve has three characteristic points: the inflection point ‘K’ where the growth changes from increasing to decreasing, the point of maximal acceleration ‘s1’, and the point of maximal deceleration ‘s2’. (B) Data and curve fits for line CS76226 (Se-0), representative for the 11% of the plants for which the growth curve reached ‘K’. The data were fitted to the three models. It is shown that even if the inflection point ‘K’ is reached, model Expo1 and Expo2, which both assume indeterminate growth, resulted in good fits. (C) Data and curve fits for line CS76308 (ZdrI2-25), representative for 56% of the plants for which the growth curve did not reach ‘s1’. The data were fitted to the three models. (D) Magnification of (C) allowing better comparison of the various models for the time window of the experiment.

To be able to quantify the dynamics in rosette growth over time, growth was modelled using different mathematical functions (Table 2; Supplementary Table S2 at *JXB* online). Determinate growth (i.e. growth that terminates before the end of the life cycle of an organism) can in many cases be described by a sigmoid function (S-curve). Rosette growth of *Arabidopsis* is known to be determinate, following such an S-curve (Boyes *et al.*, 2001). S-curves are characterized by an accelerating phase, a linear phase, and a saturation phase (Fig. 3A). Within the linear phase, which is not really linear, but can be approached by a linear function, the inflection point ‘K’ is located. At ‘K’, the curve changes from increasing growth to decreasing growth. Near the end of the acceleration phase, which can be approached by an exponential function, the point of maximal acceleration ‘s1’ is located. Near the beginning of the saturation phase, the point of maximal deceleration ‘s2’ is reached and, thereafter, the growth gradually stops and the final rosette size ‘*A*_max_’ is reached. Determinate growth was modelled using the Gompertz function (Gom), which results in an S-curve (Gompertz, 1825; Winsor, 1932) (Table 2). This function is a slightly modified form of the basic logistic function, which was first described by Pierre Verhulst in 1838 (Verhulst, 1838). The modifications of the basic logistic function change this basic symmetric growth curve into an asymmetric one. The Gompertz function used here contains three parameters: ‘*A*_max_’, the final rosette size; ‘*b*’ that determines the position of the curve along the time axis; and ‘*r*’ that determines the growth rate at the inflection point ‘K’ (Table 2). The combination of these three parameters determines on which day ‘s1’, ‘s2’, and ‘K’ are reached. As the growth curves fitted with Gom were investigated, none of the plants in this experiment reached ‘*A*_max_’ and only 4% reached ‘s2’within the window of the experiment. Even ‘K’ and ‘s1’ were not reached by the majority of the plants: for 89% of the plants ‘K’ was not reached before day 28 and for 57% of the plants ‘s1’ was not reached before day 28. This means that for most plants the collected data points are located in the accelerating phase and the beginning of the linear phase of the growth curve, implying that the plants had not yet entered the saturation phase. This was expected for the plants that had not yet bolted, but for the 30% of the plants that were bolting on the last day it was expected that they would have reached at least the saturation phase, because earlier studies reported that *Arabidopsis* rosettes reach the final size when they start to flower (Boyes *et al.*, 2001). Because most plants were in the acceleration phase even on the last day of the experiment, the growth was modelled not only with the Gompertz curve that describes determinate growth, but also with models that describe indeterminate growth (e.g. exponential growth). The simplest indeterminate growth model used was based on an exponential function (Expo1) with one parameter ‘*r*’, which represents the growth rate (Table 2). This model assumes that the growth rate is equal during the whole growth period and that the initial size (*A*_0_) is 1 (Table 2). Exponential growth was also modelled with a function (Expo2) with two parameters, growth rate ‘*r*’ and the initial size (*A*_0_) (Table 2; Supplementary Table S2). *A*_0_ not only represents the starting value, but it is also a magnification factor. This means that for two plants with equal ‘*r*’ and a factor two difference in *A*_0_, plant size is also a factor of two different during the whole experiment. To illustrate the use of the three models, data from two plants that showed determinate and indeterminate growth were used for curve fitting (Fig. 3B–D). The plant with determinate growth is representative for 11% of the plants in which growth reached ‘K’ within the course of the experiment (Fig. 3B). The plant with indeterminate growth is representative for the 56% of the plants for which growth did not reach ‘s1’ within the course of the experiment (Fig. 3C, D).

**Table 2.** Mathematical functions used to model growth and their properties

Model	Formula	Description parameters	Remarks
Expo1	PLA=*e* ^*r*×*t*^	*r*: growth rate	A_0_=1
Expo2	PLA=*A*_0_×*e* ^*r*×*t*^	*A*_0_: initial size	*A*_0_ is also a magnification factor
		r: growth rate	
Gompertz	$\text{PLA}=A_{\max}\times e^{-b\times e^{-r\times t}}$	*A*_max_: final rosette size	Limits used for fitting of data: *A*_max_=20 000, *b*=60
		*b*: position along the time axis	
		*r*: growth rate at inflection point ‘K’	

**Fig. 4.**
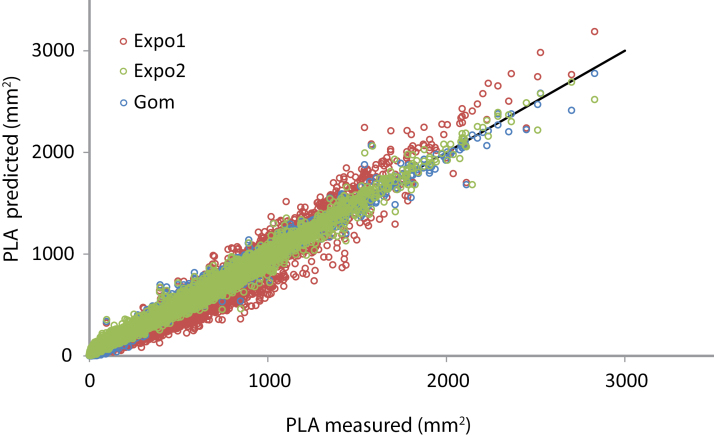
Comparison of the goodness-of-fit for the three growth models used: exponential function with one (Expo1) or two (Expo2) parameters, and Gompertz function (Gom). Plot of the measured PLA on days 8, 11, 14, 16, 18, 20, 22, 24, 25, 26, 27, and 28 against the predicted PLA on the same days. The black line represents *y*=x (PLA measured=PLA predicted).

For each model, the goodness-of-fit was evaluated (Fig. 4; Supplementary Table S2 at *JXB* online). As expected, *r* ^2^ was on average higher when more parameters were introduced into the model (Supplementary Table S2). Expo1 predictions were in general too low at small PLA and too high at large PLA (Fig. 4), which indicates that this model is too simplistic. Interestingly, the differences in goodness-of-fit between Expo2 and Gom were not large, emphasizing that most plants in this experiment do not reach ‘K’ and that the growth rate thus does not decrease significantly during the duration of the experiment. So, for most plants in this experiment, determinate growth cannot be concluded from the PLA data collected. This was supported by the smaller confidence intervals for the parameters of Expo2 compared with the parameters of Gom (Supplementary Table S2). For *A*_max_ in particular, very large confidence intervals were observed. Based on Fig. 4, the confidence intervals and the principle of parsimony, stating that the simplest of two competing models is to be preferred, Expo2 was chosen to be used in the GWA analyses. This model is counter-intuitive because it describes indeterminate growth, while it is known that the *Arabidopsis* rosette follows determinate growth (Leister *et al.*, 1999). In this case, a model describing determinate growth, such as the Gompertz function, results in parameters that are more informative (or speculative) for growth outside than inside the experimental window. Growth models that describe an S-curve always contain a parameter representing the final rosette size, and the other parameters that are estimated are dependent on that parameter. In the present case, Gom, which describes an S-curve, would have functioned as a (not very reliable) predictive model instead of a descriptive model as was aimed for. If curve fitting using the growth model results in reliable fits, as it did for most plants in this experiment, it allows for comparison of plants which differ in developmental timing, growth rate, and plant size. However, this comparison only leads to valuable insight if the right model is chosen. Conclusions based on a non-optimal model should be interpreted carefully as they can easily become very speculative (Tessmer *et al.*, 2013).

### Quantification of growth dynamics by exponential growth model parameters

Moderate to high heritabilities was observed for the growth parameters estimated by Expo2 (Table 1), indicating that these growth characteristics are determined partly by the genotype. The parameter ‘*r*’ of Expo2 is weakly correlated with plant size in week 2 and week 3, but no correlation was observed with plant size at the end of the experiment (Fig. 2). This indicates that the growth characteristic represented by this parameter goes beyond simple information about plant size. The model assumes a certain growth pattern and, given the parameters, describes exactly how this growth takes place. So the mathematical function used in the model expresses the overall shape of the growth curve shared by all the data. The details of the growth curve, determined for each individual plant, are described by a specific set of model parameters. Parameter ‘*A*_0_’, which represents the initial size of the plant, is positively correlated with plant size in weeks 2, 3, and 4. This is in accordance with the observation that the size of the plant at different time points is highly correlated throughout the whole experiment. Natural variation is observed for seed size and seed germination (Schmuths *et al.*, 2006; Vallejo *et al.*, 2010; Herridge *et al.*, 2011), and these traits have also been determined for the accessions used in this experiment (Joosen *et al.*, 2013). Correlation between *A*_0_ and PLA determined in this experiment and the seed traits reported by Joosen *et al.* (2013) is limited: only weak (*r* ^2^ between 0.1 and 0.3) but significant correlations were found for seed size (dry and imbibed), but not for germination traits. The environment in which the parental plant matures has a large impact on seed weight and germination rate (Elwell *et al.*, 2011), and therefore differences between seed batches are expected. Thus, the germination rate could have influenced plant size in the present experiment, although no correlation was found between seed germination traits measured in Joosen *et al.* (2013) and PLA and *A*_0_ determined in this experiment. A strong negative correlation was found between the two model parameters ‘*A*_0_’ and ‘*r*’ (*r* ^2^= –0.71), which can partly be explained by the fact that a relatively high value for *A*_0_ (*A*_0_>10) was never found in combination with a relatively high *r* (*r*>0.20) (Supplementary Fig. S1 at *JXB* online). This has to do with the boundaries of the natural variation. Probably, for *Arabidopsis*, too rapid growth is not favourable in nature and therefore gene combinations that would lead to both a large *A*_0_ and a large value for *r*, and hence would result in enormous plants, are not selected for. In addition, enormous plants are probably also physically not possible. Taking all these correlations into account, it can be concluded that determination of growth over time and subsequent modelling of growth results in the quantification of growth dynamics that provide insight into the growth patterns that could not have been obtained from single time point measurements.

### Added value of dynamic growth modelling in GWA mapping

**Fig. 5.**
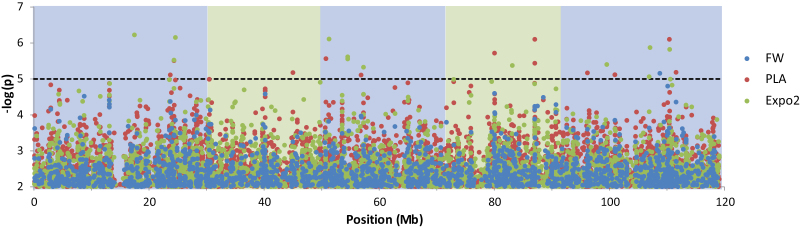
Genome-wide association (GWA) mapping of FW, PLA, and parameters of growth model Expo2. Univariate GWA analyses were performed for all traits; in addition, the model parameters ‘*r*’ and ‘*A*_0_’ were also analysed together in an MTMM-GWA approach. A Manhattan plot of the –log(*P*) marker–trait association for FW, PLA, and model parameters of Expo2 is shown. PLAs on the different days are represented by one value; for each SNP, only the –log(*P*) value of the day with the highest association is plotted. Univariate analyses of ‘*r*’ and ‘*A*_0_’, and the MTMM analyses of ‘*A*_0_’ and ‘*r*’ jointly are also represented by one value; for each SNP, only the –log(*P*) value of the analysis with the highest association is plotted. The total number of tested SNP markers was 214 000, but only the ~10 000 SNPs with –log10(*P*)>2 are plotted. The dotted line indicates the arbitrary threshold of –log(*P*)=5.

**Fig. 6.**
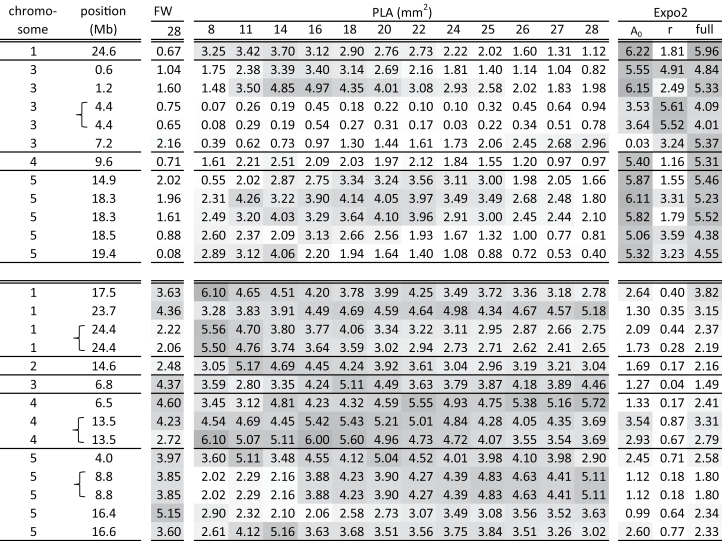
Association profiles of SNPs that were identified by GWA mapping to be highly associated with the traits FW, PLA over time, and Expo2 model parameters. Each number in the columns with heading ‘FW’ or ‘PLA (mm^2^)’ represents the association found by univariate GWA mapping of growth trait by time point as indicated at the top of the column (FW at day 28 or PLA on one of the indicated days) and the SNP at the position indicated in the first two columns. In the last three columns, with the heading ‘Expo2’, the numbers indicate the association found between SNPs and parameters of model Expo2. Columns with the heading ‘Expo2: A_0_’ and ‘Expo2: r’ refer to univariate GWA mapping of model parameters ‘*A*_0_’ and ‘*r*’ respectively, and the column with heading ‘Expo2: full’ refers to multivariate GWA mapping of both growth model parameters. All SNPs with –log(*P*)>5 for at least one trait are shown. The intensity of the grey colour corresponds to the strength of the association. Curly brackets indicate that associated SNPs are located within 10kb and are considered as one QTL.

To identify the genetic basis of growth, GWA mapping was performed on PLA data (12 different dates), FW data (end point), and on the parameters derived from the selected growth model Expo2 (Fig. 5). Parameters of models with fits of *r* ^2^<0.9 (11 out of 965) were excluded to avoid bias in detected associations due to outliers created by poor fits. PLA, FW, and model parameters were mapped as independent traits, even though they display covariance. The two parameters of Expo2, ‘*r*’ and ‘*A*_0_’, were also mapped simultaneously using an MTMM approach, which takes covariance of parameters into account (Korte *et al.*, 2012). In total, 26 SNPs were highly associated [–log(*P*)>5] with one or more of the traits. One of these SNPs was associated with FW, 13 SNPs were associated with PLA, and 12 SNPs were associated with the model parameters. For each of these 26 strongly associated SNPs, an association profile was made to identify whether associations were specific for a trait or time point, or whether they were more general (Fig. 6). SNPs displaying a profile with strong associations for FW and PLA over time were not or only moderately associated with the Expo2 parameters (Fig. 6B). For example, the association profile of two SNPs at chromosome 5 at 8.8Mb was moderate to high for PLA at weeks 3 and 4 [–log(*P*) between 3.88 and 5.11], moderate for FW [–log(*P*)=3.85], and low for model parameters [–log(*P*)<2]. This trend was also observed conversely, although some SNPs that were highly associated with model parameters were also found to be moderately to highly associated with PLA at some time points (Fig. 6). For example, the association profile of the SNP at chromosome 3 at 1.2Mb that was high for parameter ‘*A*_0_’ [–log(*P*)=6.15] and for the multitrait analysis of ‘*r*’ and ‘*A*_0_’ [–log(*P*)=5.33] was also high for PLA in the third week [–log(*P*) between 4.01 and 4.97]. Remarkably, SNPs that were highly associated with model parameters were never associated with FW at day 28. This emphasizes that the model parameters reveal characteristics of growth that would not have been detected if only final plant size data were considered. Growth modelling, therefore, resulted in the detection of QTLs that would not have been found otherwise. Nonetheless, GWA mapping of model parameters cannot replace GWA mapping of plant size data, because both methods resulted in the detection of unique, highly associated, SNPs. SNPs that were selected because of strong association with PLA at a specific time point had an association profile for PLA that was relatively high [–log(*P*)>2] during the whole course of the experiment. This observation is in accordance with the significant positive correlation between PLA at different time points throughout the experiment (Fig. 2). These data indicate that the growth phenotype of a plant is the result of the interplay of many different genes and that the composition or contribution of this set of growth factors will change during the development of the plant. Some genes only play a role at a specific time point, whereas other more general regulators may have a function in growth for a longer period. Many transcription factor are, for example, known to be expressed in both a developmental time-specific and a tissue-specific manner (Turnbull, 2011), whereas their influence on plant development is visible during several developmental stages and, in other tissues, due to the expression of downstream targets. Similarly, levels of phytohormones are tightly regulated over time, whereas prolonged downstream effects are often observed (Schachtman and Goodger, 2008). The relative effect size of these regulators might change over time as a result of the dynamic balance between different regulatory components during development. The effect of these general growth factors will, therefore, only be large enough at specific time periods to be detectable with GWA mapping. SNPs that were selected because of strong association with PLA at a specific time point may, therefore, point to genes that play a role at a very specific period of development, but they may also point to more general regulators. If plant size had only been measured at one time point, many of these time-specific associations would not have passed the threshold, and thus would have been missed. Most striking is the observation that only one SNP was strongly associated [–log(*P*)>5] with FW at day 28, so if growth was only evaluated by end-point FW determination, all except one of the associations would have been missed. Thus, the analyses therefore show that evaluation of growth over time is more powerful to identify the underlying genetic factors than the evaluation of growth by end-point measurements. This is especially true when many small effect genes, whose relative contribution may change over time, are underlying the trait of interest.

### Novel candidate genes for growth dynamics

Because GWA mapping was done in a natural population of accessions with strong linkage disequilibrium (LD) decay, causal genes are expected to be located in close proximity to the associated SNPs. Because the LD decay in *Arabidopsis* is on average 10kb (Kim *et al.*, 2007), significant SNPs that were located within this distance from each other were considered to be associated with the same causal gene. This approach resulted in 11 QTLs for the model parameters and another 11 QTLs for FW and PLA over time (Fig. 6). Genes that were located within a support interval of 10kb upstream and downstream from these QTLs were selected for further analyses (Table 3; Supplementary Table S3 at *JXB* online). It is known that LD decay is not equal along the whole genome and that the causal gene can, therefore, be located outside the 10kb window. However, even in studies of linkage mapping in recombinant inbred line (RIL) populations, the confirmed causal genes were in most cases located very close to the associated marker even when the support interval was large (Price, 2006). For QTLs represented by multiple SNPs in close proximity, the support window was taken 10kb upstream of the first SNP to 10kb downstream of the last SNP, therefore candidate genes in such QTLs could be located >20kb apart. For example, the two associated SNPs on chromosome 4 at 13.5Mb are located 6.3kb from each other, so candidate genes for this QTL can be located 26.3kb from each other. Large differences were observed in gene density in the support windows, ranging from two up to 12 genes (Table 3). As expected, the number of genes was in general higher in the support window of QTLs that represent multiple associated SNPs. In total, 97 genes were selected, 41 for QTLs associated with model parameters and 56 for QTLs associated with plant size.

**Table 3.** *Information about the support window around the 26 SNPs that are highly associated with the growth traits [–log*(P*)>5]* The order of SNPs corresponds to those in Fig. 6 to enable easy comparison of the data presented. Bold indicates that associated SNPs are located within 10kb and can be considered as one QTL.

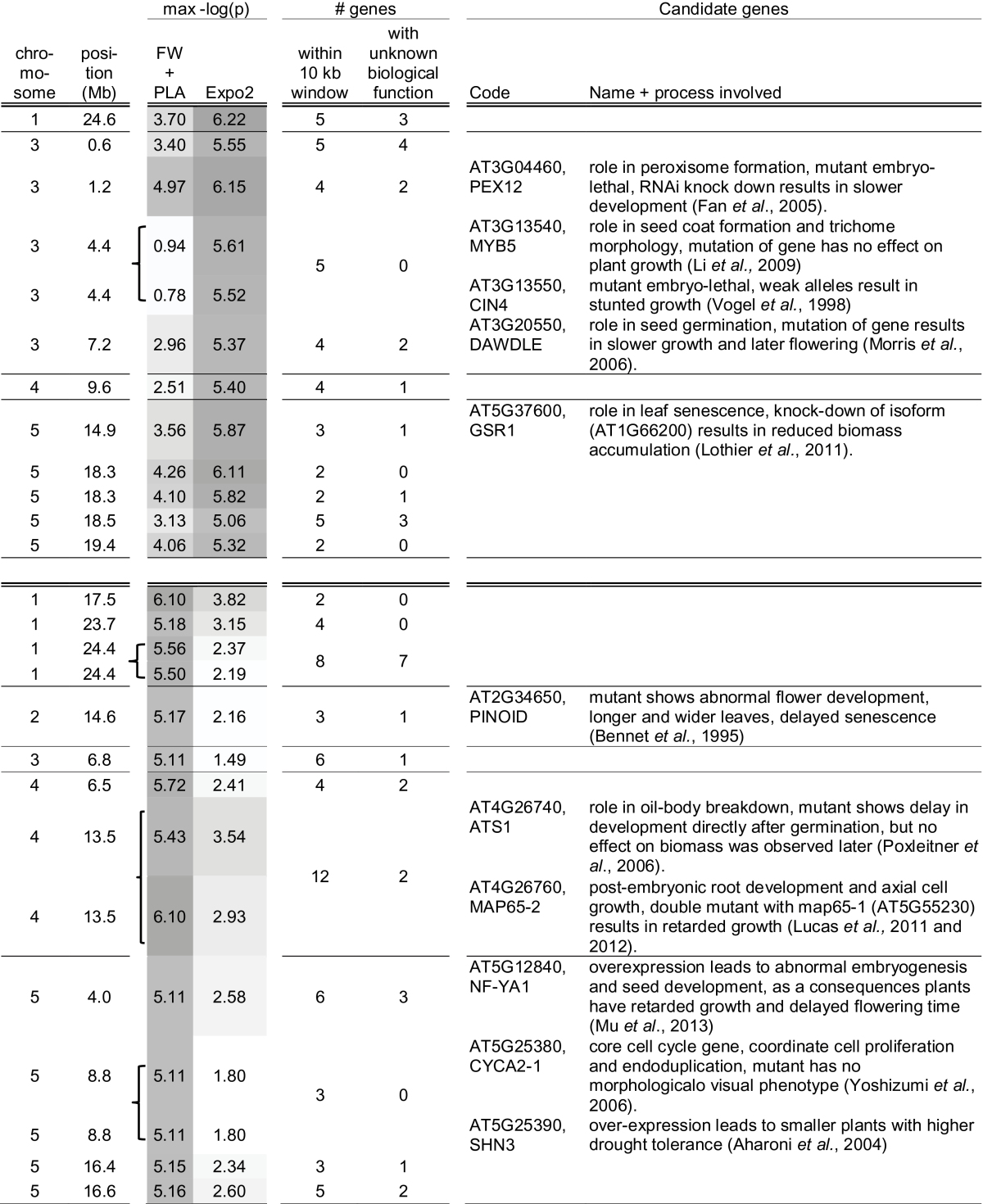

The annotation of the 97 genes within the support windows was analysed and the 17 genes with GO terms ‘developmental processes’ and the 13 genes with GO term ‘transcription’ (TAIR10) were studied in more detail. Both GO terms were not significantly over-represented within the candidate genes (Plant GSEA; Yi *et al.*, 2013). Over-representation is not expected for common GO terms, because only one or a few causal genes at most are expected per QTL and therefore only a limited number of the 97 candidates genes will be causal. All other genes are presumed to be randomly distributed over GO categories. The QTL on chromosome 4 at 13.5Mb illustrates the significance of the present findings. This QTL contains two SNPs that are highly associated with plant size in weeks 2 and 3, and moderately associated with plant size in week 4 (Fig.e 6). A weak association with these SNPs was also found in the univariate GWA mapping of parameter ‘*A*_0_’ and the multivariate analyses of ‘*A*_0_’ and ‘*r*’. Within the support interval of this QTL, 12 genes are located, only two of which are annotated to be involved in plant development and none is a transcription factor. The first gene, AT4G26760 (MAP65-2), is involved in post-embryonic root development (Lucas and Shaw, 2012) and axial cell growth in hypocotyls (Lucas *et al.*, 2011). This gene is most closely located to one of the associated SNPs, at only 2.1kb. Double mutants of this gene and the closest homologue AT5G55230 (MAP65-1) show retarded growth, and therefore AT4G26760 is a strong candidate for the causal gene underlying this QTL. The second gene, AT4G26740, is only expressed in the embryo and plays a role in the breakdown of oil bodies. Directly after germination, mutants show a delay in growth, but this does not affect biomass accumulation at a later stage and, therefore, this gene is less likely to be the causal gene. However, a role in natural variation of plant size cannot be excluded for this gene, because no accessions other than Columbia have been analysed, and redundancy in function may be present (Briggs *et al.*, 2006). Two other genes in the region encode unknown proteins, and the remaining genes play roles in processes that are not directly linked to biomass accumulation. To confirm that AT4G26760 is responsible for the observed natural variation in growth, it is necessary to perform experiments in which more accessions other than Columbia are investigated. Exchange of alleles between natural accessions can be a powerful tool to identify allele-specific growth phenotypes. The QTL on chromosome 1 at 24.6Mb demonstrates that follow-up research of GWA mapping is not straightforward. This QTL contains the strongest associated SNP identified in this experiment [–log(*P*)=6.22]. Strong association was found for univariate GWA mapping of parameter ‘*A*_0_’ and the multivariate analyses of ‘*A*_0_’ and ‘*r*’, and weak associations were found with PLA in weeks 2 and 3. Three genes with unknown function and two genes related to defence are located in the support interval. Additional information about gene expression or the phenotypes of mutant or overexpression lines is needed to be able to prioritize these genes and finally confirm the causal gene. This is a laborious and time-consuming process, which is probably the reason why not very many non-obvious candidates have been confirmed in GWA mapping as yet.

Because a weak relationship was noticed between growth rate and flowering time, the candidate gene list was also screened for genes involved in the regulation of flowering. Only one of the 97 candidate genes was related to flowering time (Table 3; Table S3 at *JXB* online). Mutants of this gene [i.e. DAWDLE (AT3G20550)] show delayed flowering and slower growth (Morris *et al.*, 2006). This corresponds to the observation that plants that bolted within the experimental time were larger than those that had not yet bolted. DAWDLE stabilizes the hairpin formation of microRNAs (miRNAs; Yu *et al.*, 2008). Three miRNAs influence the expression of FT and SOC1 (Yamaguchi and Abe, 2012), two key players in the flowering time regulatory network downstream of FLC. The SNP associated with DAWDLE (chromosome 3, 7.2Mb) was only identified in the MTMM analyses of *A*_0_ and *r* simultaneously, which is probably the reason why this gene was not identified previously in any RIL or GWA mapping study regarding flowering time or biomass. Atwell *et al.* (2010) found a strong association between flowering and FLC using a natural population of 95 accessions, of which four are overlapping with the present set, confirming allelic differences for FLC between natural accessions (Gazzani *et al.*, 2003; Guo *et al.*, 2012). In the present study, a weak association [–log(*P*)=3.19] was found between plant size on day 27 and an SNP (chromosome 5 pos 95343751) in LD with SNPs in FLC. However, the population is very diverse and, consequently, sequence variation for many other flowering time genes is expected. This might be the reason why the association between FLC and plant size is not stronger.

For one-third of the genes within the support windows, no biological function has been annotated (34 genes, TAIR10). GWA mapping is an approach that is not hypothesis driven but rather data driven. It aims to find genes that contribute to the explanation of variation observed for a trait, without the need to know the pathway or mechanism by which the phenotype and the genotype are correlated. GWA mapping is a powerful method to find novel functions for genes, or to identify functions of unknown genes. Unfortunately, such new functions have hardly been reported yet, because most studies that report associations identified by GWA mapping are not coupled with studies to confirm candidate genes. Therefore, the attention in GWA mapping studies is biased towards genes whose functions are already known, and the genes with unknown biological functions are given less attention. However, in the field of plant sciences, a report of the confirmation of an unknown gene identified by GWA mapping was recently published (Meijon *et al.*, 2014) and hopefully many publications will follow soon.

In summary, considering all 97 candidate genes, 11 are annotated to play a role in the determination of cell number, cell size, seed germination, embryo development, transition from the vegetative to generative stage, or senescence. For eight of these genes, a mutant or overexpression phenotype related to growth has been reported. These eight genes are located in the support window of eight QTLs, four of which were associated with model parameters and four with plant size (see details in Table 3). This emphasizes that mapping of growth model parameters is complementary to the mapping of plant size data at several time points separately. For none of the eight candidates are growth dynamics reported, and it is therefore not known whether allelic variants affect growth from the start, only in a specific developmental stage, or from a specific developmental stage onwards. Therefore, additional temporal growth and gene expression data need to be collected to determine whether the candidate genes play a time-specific or a general role in plant growth regulation. For none of the genes in the support window of the other 13 QTLs has a mutant or overexpression phenotype related to biomass accumulation been reported yet. These findings indicate that the observed associations are likely to be true positives and that many more genes are involved in growth regulation than are currently known.

### Conclusions

Here, a series of analyses are described that started with the observation of growth dynamics by automatic imaging and that, by subsequent image analysis, growth modelling, and GWA mapping, resulted in the indication of candidate genes involved in growth regulation. Top-view imaging of *Arabidopsis* plants in combination with high-throughput image analysis allowed rosette growth to be followed over time in a large and diverse population of natural accessions. During the experiment, most rosettes were in the acceleration and linear phase of growth, which could be modelled best by an exponential function (Expo2) describing indeterminate growth. Modelling ensured proper comparison of the diverse panel of accessions demonstrating large variations in the rate of development and in plant size. To identify the genetic basis of growth, GWA mapping was performed on PLA data (12 different dates) and FW data (end-point), and on the parameters derived from the growth model Expo2. This resulted in the detection of 22 growth QTLs which were highly associated [–log(*P*)>5] with the growth traits. Many of these QTLs would not have been identified if growth had only been evaluated at a single time point. Eight candidate genes were identified for which a mutant or overexpression phenotype related to growth has previously been reported, suggesting that the identified QTLs are true positives. For some QTLs, no obvious candidates were found, opening up the way to identify new functions for underlying genes or to annotate unknown underlying genes by performing follow-up experiments.

## Supplementary data

Supplementary data are available at *JXB* online.

Figure S1. Scatter plot of the Expo2 model parameters ‘*A*_0_’ and ‘*r*’.

Table S1. Projected leaf area, fresh weight, model parameters of Expo2, water content, and bolting at day 28 of 324 natural accession of *Arabidopsis* grown in the PHENOPSIS Phenotyping platform in three replicates.

Table S2. Model parameters, 95% confidence intervals, and goodness-of-fit data of three replicates of 324 natural accession of *Arabidopsis*.

Table S3. TAIR10 gene description of the 97 candidate genes.

Supplementary Data
