# Supplementary material for: Genome-wide association mapping of growth dynamics detects time-specific and general quantitative trait loci
Source: J Exp Bot. 2015 Apr 28;66(18):5567–80. doi: 10.1093/jxb/erv176 (PMC4585414; doi:10.1093/jxb/erv176)
Supplement: Supplementary Data [file supp_66_18_5567__index.html]

Genome-wide association mapping of growth dynamics detects time-specific and general quantitative trait loci — Genome-wide association mapping of growth dynamics detects time-specific and general quantitative trait loci — Supplementary Data 

# Genome-wide association mapping of growth dynamics detects time-specific and general quantitative trait loci

## Supplementary Data

Data files

**Files in this Data Supplement:**

- Supplementary Data - Supplementary Data
- Supplementary Data - Supplementary Data
- Supplementary Data - Supplementary Data
- Supplementary Data - Supplementary Data
