# Supplementary material for: Genome-wide association mapping of growth dynamics detects time-specific and general quantitative trait loci
Source: J Exp Bot. 2015 Apr 28;66(18):5567–80. doi: 10.1093/jxb/erv176 (PMC4585414; doi:10.1093/jxb/erv176)

Figure S1 Scatter plot of the Expo2 model parameters 'A0' and 'r'. Area of the bubbles corresponds with the FW of the rosette at day 28. **A** Scatter plot containing data of all individual plants in the experiment. **B** Zoom as indicated in A.

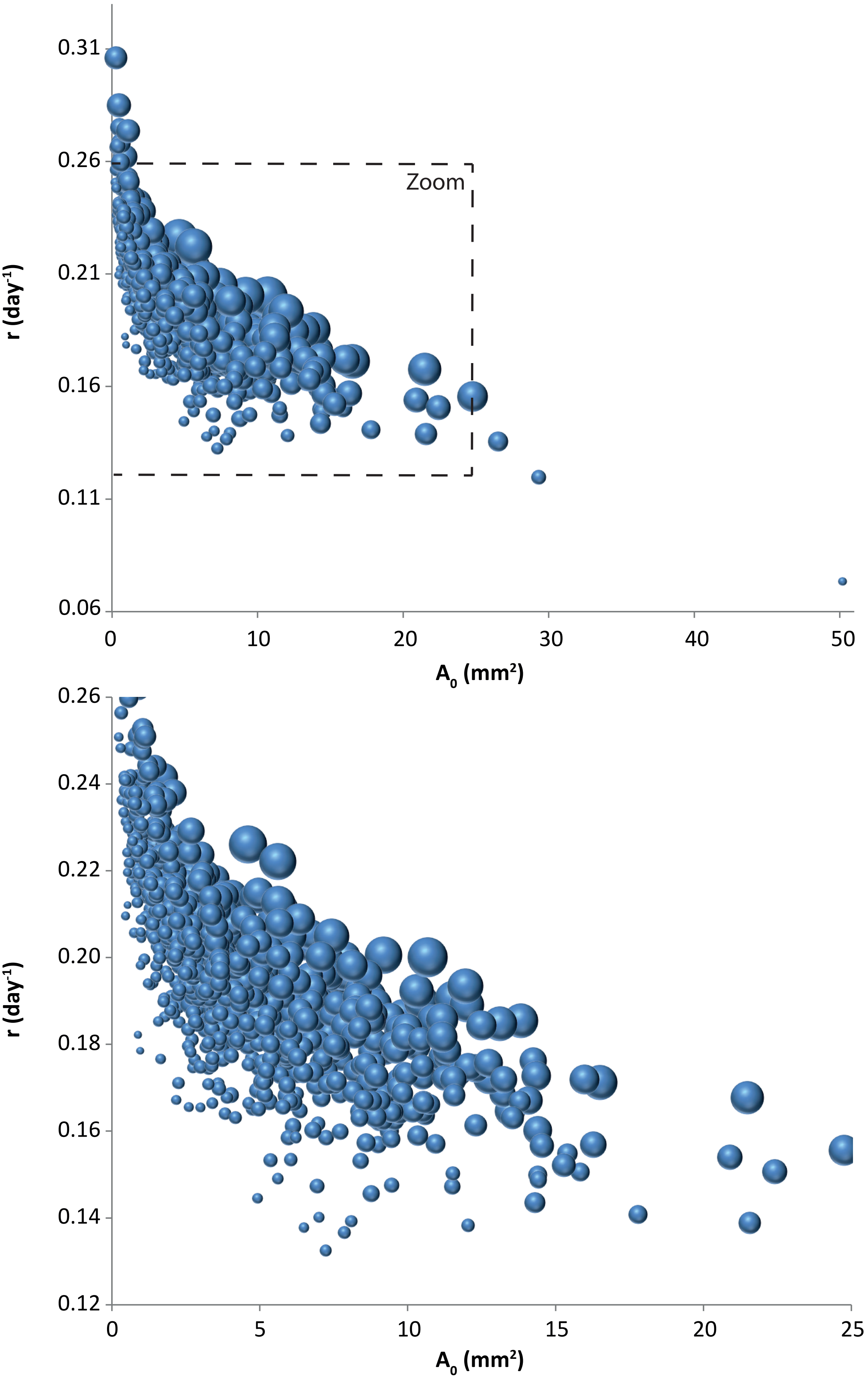

Supplement: Supplementary Data [file supp_erv176_jexbot139626_file004.pdf]
